# Histopathological Spectrum of Ovarian Neoplasms: A Single-Center Study

**DOI:** 10.7759/cureus.27486

**Published:** 2022-07-30

**Authors:** Ayma Batool, Zonaira Rathore, Fizza Jahangir, Saira Javeed, Saira Nasir, Akhtar S Chughtai

**Affiliations:** 1 Histopathology, Chughtai Institute of Pathology, Lahore, PAK

**Keywords:** who, sex cord-stromal tumors, germ cell tumors, surface epithelial tumors, ovarian cancer

## Abstract

Introduction

Among female genital tract-related malignancies, ovarian tumors are the leading cause of mortality. The present study was conducted to identify the various histopathological spectrums of ovarian neoplasm (ON) according to the World Health Organization (WHO) classification in a single center.

Material and methods

This cross-sectional study was conducted over a period of one year (November 2020 to October 2021) in the Department of Histopathology, Chughtai Institute of Pathology (CIP), Lahore, Pakistan. The study included 390 cases of ovarian neoplasms (ONs). After proper fixation and thorough gross examination, sections were routinely processed and examined. The distribution of the various histomorphological spectrum of ovarian tumors was studied according to the WHO classification.

Results

Out of the 390 cases studied, 320 (82.05%) were benign, 11 (2.82%) borderline, 57 (14.61%) malignant, and two (0.52%) metastatic tumors. Surface epithelial tumors (SETs) (246 (63.08%)) were the most common of all, followed by germ cell tumors (GCTs) (115 (29.48%)) and sex cord-stromal tumors (SSTs) (27 (6.92%)). The largest percentage (36.15%) of ONs was seen in 21-30 years of age group.

Conclusion

The present study shows various histopathological features of ONs. Benign tumors are more common than malignant tumors. Among the benign tumors, serous tumors were most common of all. Thus, an accurate histological diagnosis is important to initiate a proper management plan.

## Introduction

Ovarian neoplasms (ONs) comprise 3% of all cancers [1,2], and among female genital tract-related malignancies, the incidence is 25% [3,4]. Ovarian cancer is the fifth most prevalent cancer overall and the second most common gynecological cancer [3,5]. Non-Hispanic whites (11.6 per 100,000) have the highest rate of ONs, followed by the second highest frequency in American Indians and Alaska Natives (10.3 per 100,000), Hispanics (10.1 per 100,000), non-Hispanic blacks, and Asian and Pacific Islanders [6].

Ovaries are paired organs in the female reproductive system which can undergo various changes throughout an individual’s life under the effects of different hormones. This could lead to different types of diseases, benign or malignant [2,7]. Most of the ONs are benign and usually diagnosed at 30-40 years of age, while malignant tumors are diagnosed at 60-70 years of age [7]. Ovarian cancer has the worst prognosis among gynecological malignancies due to a lack of proper signs, symptoms, and presentation and is usually detected in the later stages of the disease. Most ONs occur during the reproductive age group. Nulliparity, a high socioeconomic status, and environmental and genetic factors are all important risk factors [8]. ONs cannot be diagnosed alone on the basis of clinical, radiological, and gross findings. Hence, a precise histopathological diagnosis is required for starting definitive treatment [2,9]. The present study was conducted with the aim to evaluate the histopathological spectrum of ONs in a single center in Lahore, Pakistan.

## Materials and methods

The present cross-sectional study was conducted over a period of one year (November 2020 to October 2021) at the Chughtai Institute of Pathology (CIP), Lahore, Pakistan, after obtaining approval from the institutional review board (letter no. CIP/IRB/1053). A total of 390 cases were included in the study. The specimen was received and fixed in 10%/formalin. After proper fixation of 24-48 hours, representative sections were taken and routinely processed with paraffin embedding. Tissue sections of four to five microns were cut using a microtome and stained with hematoxylin and eosin. The slides were reviewed by two pathologists having a special interest in female genital tract pathology, and the tumors were classified according to the WHO classification of ovarian tumors.

Data were analyzed using SPSS, version 22 (IBM Corp, Armonk, NY, USA). The large data were sorted into various datasets based on the distribution of tumors as surface epithelial tumors (SETs), germ cell tumors (GCTs), sex cord-stromal tumors (SSTs), and metastatic tumors. The patients were divided into different age groups, and the frequency of ONs was analyzed.

Inclusion criteria

All ovarian neoplasms and all age groups were included.

Exclusion criteria

Non-neoplastic ovarian lesions and poorly fixed and autolyzed specimen were excluded.

## Results

The present study included a total number of 390 cases of ONs. The age range lies between 10 and 80 years (Table 1), and ovarian tumors were classified according to the WHO classification (Table 2). Benign tumors (320 (82.05%)) were more common than malignant ones (57 (14.61%)). Borderline tumors comprised 11 (2.82%) and metastatic tumors two (0.52%) out of all ON cases. SETs were the most common of all (Table 3), followed by GCTs (Table 4), while SSTs comprised a total of 27 (6.92%) cases (Table 5).

**Table 1:** Age-Wise Distribution of Ovarian Neoplasms

Age (years)	Surface epithelial tumors	Germ cell tumors	Sex cord-stromal tumors	Metastatic tumors	Total number
11-20	11	12	0	0	23
21-30	79	57	5	0	141
31-40	85	30	7	0	122
41-50	30	15	9	0	54
51-60	20	1	5	0	26
61-70	13	0	1	2	16
>70	8	0	0	0	8
Total	246	115	27	2	390

**Table 2:** Distribution of Ovarian Neoplasms According to the WHO Classification

Type	Number	Percentage (%)
Surface epithelial tumors	246	63.08
Germ cell tumors	115	29.48
Sex cord-stromal tumors	27	6.92
Metastatic tumors	2	0.52
Total	390	100

**Table 3:** Distribution of Surface Epithelial Tumors

Type of tumor	Benign, n (%)	Borderline, n (%)	Malignant, n (%)	Total, n (%)
Serous	118 (30.25)	3 (0.76)	28 (7.17)	149 (38.02)
Mucinous	72 (18.46)	8 (2.05)	4 (1.02)	84 (21.53)
Endometrioid	0 (0)	0 (0)	2 (0.51)	2 (0.51)
Brenner	6 (1.53)	0 (0)	0 (0)	6 (1.53)
Clear cell	0 (0)	0 (0)	5 (1.28)	5 (1.28)
Seromucinous	0 (0)	0 (0)	0 (0)	0 (0)
Total No. of cases (%)	196 (50.25)	11 (2.82)	39 (10.00)	246 (63.08)

**Table 4:** Distribution of Germ Cell Tumors

Type of tumor	Number	Percentage (%)
Mature teratoma	108	27.69
Immature teratoma	1	0.25
Mixed germ cell tumor	0	0
Dysgerminoma	3	0.76
Yolk sac tumor	2	0.51
Embryonal carcinoma	0	0
Choriocarcinoma	0	0
Monodermal teratomas (struma ovarii)	1	0.25
Gonadoblastoma	0	0
Total	115	29.48

**Table 5:** Distribution of Sex Cord-Stromal Tumors

Tumor type	Number	Percentage (%)
Fibroma	10	2.56
Thecoma	5	1.28
Juvenile granulosa cell tumor	3	0.76
Adult granulosa cell tumor	7	1.79
Sertoli-Leydig cell tumor	2	0.51
Total	27	6.92

## Discussion

The morphological and cytological characteristics of tumor cells are used to classify ONs [10]. Given their asymptomatic nature, late onset of symptoms, and lack of effective screening facilities, ovarian cancers are known as the “silent killer” [11,12]. Ovarian tumors were categorized according to the WHO classification of ovarian tumors into SETs, SSTs, GCTs, and metastatic tumors. On further subcategorization, SETs comprised serous, mucinous, endometrioid, clear cell, seromucinous, and Brenner tumors (benign, borderline, and malignant categories). GCTs comprised mature cystic teratoma, immature teratoma, dysgerminoma, yolk sac tumor, embryonal carcinoma, choriocarcinoma, mixed germ cell tumors, monodermal teratomas (struma ovarii and strumal carcinoid), and gonadoblastoma. Finally, SSTs included fibroma, thecoma, juvenile granulosa cell tumor, adult granulosa cell tumor, and Sertoli-Leydig cell tumor.

In the present study, a total of 390 cases of ONs were analyzed, out of which SETs were the most prevalent, comprising 246 cases (63.08%), GCTs 115 (29.48%), SSTs 27 (6.92%), and metastatic tumors two (0.52%). The majority were of benign category, i.e., 320 (82.05%), followed by malignant tumors 57 (14.61%). The borderline category included 11 (2.82%) cases, and two (0.52%) were metastatic tumors.

**Figure 1:**
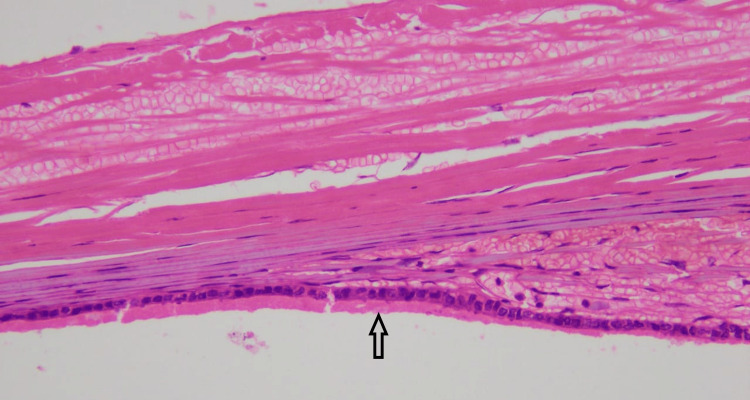
Serous cystadenoma showing cuboidal epithelium (arrow) (H&E x200) H&E: hematoxylin and eosin.

**Figure 2:**
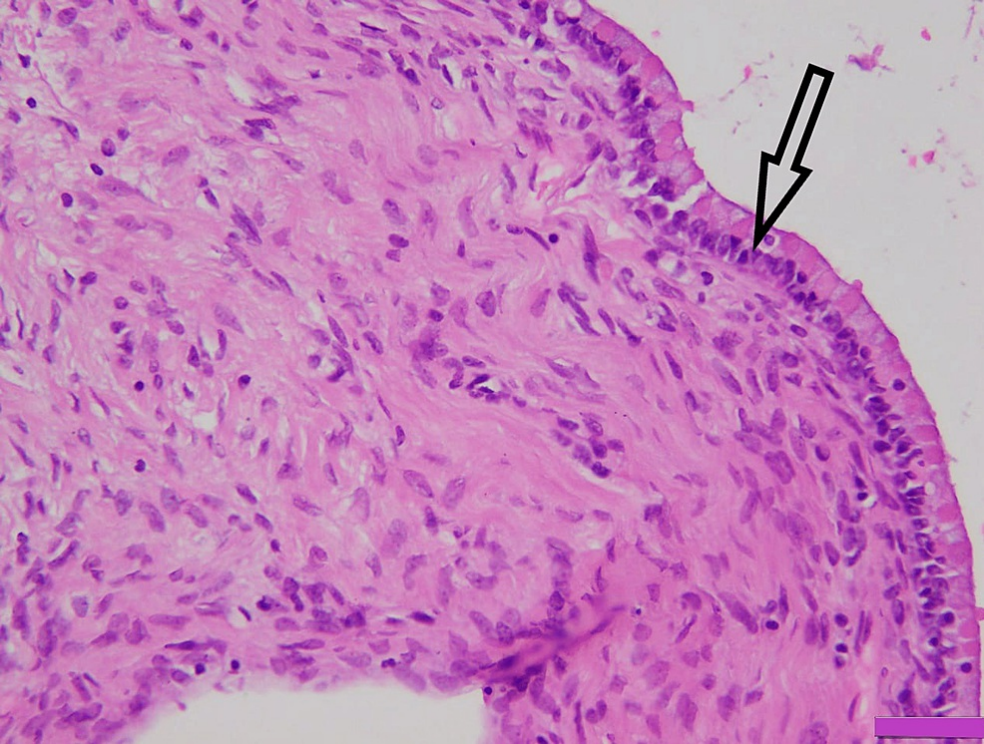
Mucinous cyst adenoma showing columnar epithelium with apical mucin (arrow) (H&E x400) H&E: hematoxylin and eosin.

The findings of our study are consistent with two other studies [2,13]. Regarding SETs, serous tumors were the most common, comprising 149 (38.2%). As indicated in Figure 1, benign serous tumors accounted for 118 (30.2%), borderline three (0.7%), and malignant 28 (7%) in the current study. The second most prevalent category among the SETs was mucinous tumors, comprising 84 cases (21.5%) (Figure 2). Brenner, clear cell, and endometrioid tumors were also diagnosed, comprising six (1.5%), five (1.2%), and two (0.51%), respectively. In our study, all the Brenner tumors belonged to the benign category, whereas the endometrioid and clear cell tumors were of malignant nature. No single seromucinous tumor was diagnosed. Almost similar results were shown by several other studies [3,14-16].

**Figure 3:**
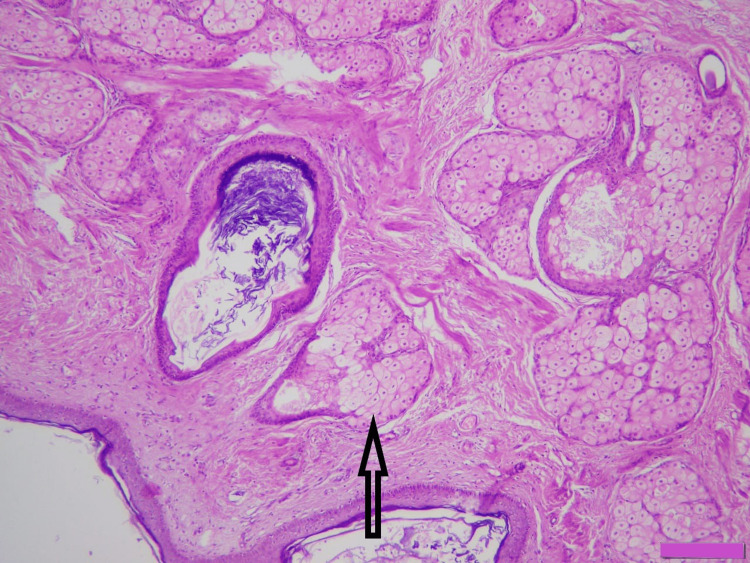
Mature cystic teratoma showing sebaceous gland (arrow) (H&E x200) H&E: hematoxylin and eosin.

**Figure 4:**
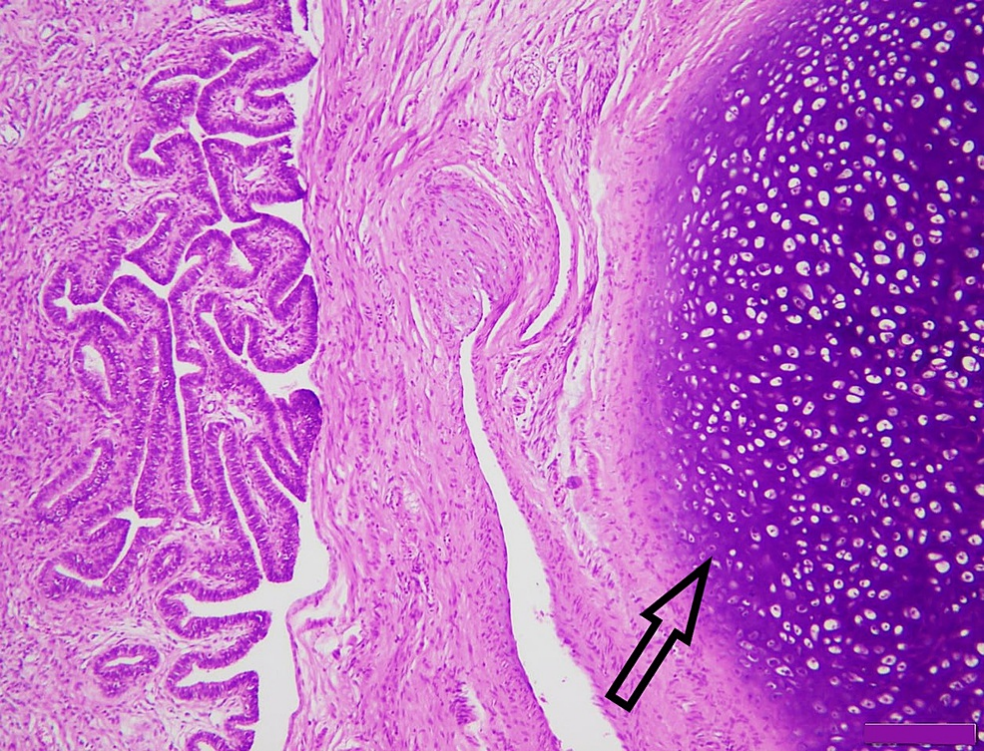
Mature cystic teratoma showing benign cartilage (arrow) (H&E x200) H&E: hematoxylin and eosin.

GCTs rank the second most common ON with a total number of 115 cases (29.48%) [3], out of which mature cystic teratoma was the most common (108 (27.6%)) as illustrated in Figures 3, 4. These findings were consistent with the previous studies [2,17,18]. No case of mixed germ cell tumor, embryonal carcinoma, choriocarcinoma, or gonadoblastoma was found.

In the present study, 27 cases (6.92%) of SSTs were included. Fibroma cases were 10 (2.56%) and accounted for a majority of them, followed by adult granulosa cell tumors, seven (1.79%). Our results were nearly identical to those of Bhagyalakshmi et al. [19] and Thakkar and Shah [20]. Metastatic tumors constituted two (0.52%) out of all ON cases. In the current study, most of the women affected were in the age range of 21-30 years, which is similar to another study conducted by Jain et al. at the Department of Pathology in Gandhi Medical College, India, on a sample size of 541 cases [21]. SET was the most common in the 31-40 years of age group, GCT in the 21-30 group, SST in the 41-50 group, and both metastatic tumors in the 61-70 group. The limitations to our study include a lack of clinical follow-up to see the prognosis of ONs. Further, as this was a single-center study in the regional laboratory of Lahore, it may not reflect the actual frequency of ONs in the whole country.

## Conclusions

The current study demonstrates a wide range of histopathological spectrum of ONs. Overall, benign tumors were more common than malignant tumors; in particular, SETs were the most common neoplasm, followed by GCTs. Serous cystadenoma was the most frequent neoplasm in the benign category, and serous carcinoma was in the malignant category. The majority of the cases were present in the 21-30 years of age group. ONs require an accurate diagnosis to be treated effectively.
